# Risk of Death and Cardiovascular Events in Asian Patients With Atrial Fibrillation and Chronic Obstructive Pulmonary Disease: A Report From the Prospective APHRS Registry

**DOI:** 10.1161/JAHA.123.032785

**Published:** 2024-03-27

**Authors:** Tommaso Bucci, Giulio Francesco Romiti, Alena Shantsila, Wee‐Siong Teo, Hyung‐Wook Park, Wataru Shimizu, Bernadette Corica, Marco Proietti, Hung‐Fat Tse, Tze‐Fan Chao, Frederick Frost, Gregory Y. H. Lip, the Asia‐Pacific Heart Rhythm Society Atrial Fibrillation Registry Investigators, Chun‐Wah Siu David, Wataru Shimizu, Kenji Yodogawa, Hiroyuki Tsutsui, Yasushi Mukai, Hirofumi Tomita, Daisuke Horiuchi, Joji Hagii, Kazutaka Aonuma, Yasuo Okumura, Masahiko Goya, Kenzo Hirao, Nobuhisa Hagiwara, Atsushi Suzuki, Teiichi Yamane, Takanori Ikeda, Hitomi Yuzawa, Kazuhiro Satomi, Yoshinao Yazaki, Keiichi Fukuda, Yoshinori Kobayashi, Norishige Morita, Toyoaki Murohara, Eiichi Watanabe, Masahide Harada, Satoru Sakagami, Takahiro Saeki, Kengo Kusano, Koji Miyamoto, Shinsuke Miyazaki, Hiroshi Tada, Koichi Inoue, Nobuaki Tanaka, Yukihiro Koretsune, Haruhiko Abe, Yasuki Kihara, Yukiko Nakano, Akihiko Shimizu, Yasuhiro Yoshiga, Tomohiro Sakamoto, Ken Okumura, Naohiko Takahashi, Tetsuji Shinohara, Kyoko Soejima, Masahiko Takagi, Mitsuharu Kawamura, Yumi Munetsugu, Sung‐Hwan Kim, Jae‐Min Shim, Jae Sun Uhm, Sung Il Im, Hyoung‐Seob Par, Jun Hyung Kim, Young Keun On, Il‐Young Oh, Seung Yong Shin, Jum Suk Ko, Jun Beom Park, Wee‐Siong Teo, Kelvin Cheok‐Keng Wong, Toon‐Wei Lim, David Foo, Shih‐Ann Chen, Shih‐Ann Chen, Tze‐Fan Chao, YennJiang Lin, Fa‐Po Chung, Yu‐Feng Hu, Shil‐Lin Chang, Ta‐Chuan Tuan, Jo‐Nan Liao, Cheng‐Hung Li, Jin‐Long Huang, Yu‐Cheng Hsieh, Tsu‐Juey Wu, Ying‐Chieh Liao, Cheng‐Hung Chiang, Hsiang‐Chiang Hsiao, Tung‐Chen Yeh, Wei‐Siang Lin, Wen‐Yu Lin, Jen‐Yuan Kuo, Chong‐Lie Hong, Yih‐Je Wu, Ying‐Siang Li, Jui‐Peng Tsai, Kuo‐Tzu Sung, Sheng‐Hsiung Chang

**Affiliations:** ^1^ Liverpool Centre of Cardiovascular Science at University of Liverpool Liverpool John Moores University and Liverpool Heart & Chest Hospital Liverpool UK; ^2^ Department of General and Specialized Surgery Sapienza University of Rome Rome Italy; ^3^ Department of Translational and Precision Medicine Sapienza University of Rome Rome Italy; ^4^ Department of Cardiology National Heart Centre Singapore Singapore; ^5^ Department of Cardiovascular Medicine Chonnam National University Hospital Gwangju Korea; ^6^ Department of Cardiovascular Medicine Nippon Medical School Tokyo Japan; ^7^ Department of Clinical Sciences and Community Health University of Milan Milan Italy; ^8^ Division of Subacute Care IRCCS Istituti Clinici Scientifici Maugeri Milan Italy; ^9^ Department of Medicine, School of Clinical Medicine; Queen Mary Hospital The University of Hong Kong Hong Kong SAR China; ^10^ Institute of Clinical Medicine, and Cardiovascular Research Center National Yang Ming Chiao Tung University Taipei Taiwan; ^11^ Division of Cardiology, Department of Medicine Taipei Veterans General Hospital Taipei Taiwan; ^12^ Danish Center for Health Services Research, Department of Clinical Medicine Aalborg University Aalborg Denmark

**Keywords:** all‐cause death, atrial fibrillation, beta blockers, COPD, heart failure, Cardiovascular Disease, Atrial Fibrillation

## Abstract

**Background:**

Chronic obstructive pulmonary disease (COPD) is associated with an increased risk of adverse events in patients with atrial fibrillation (AF); however, few data are available on this topic in Asian populations.

**Methods and Results:**

Prospective observational study conducted on patients with AF enrolled in the Asia‐Pacific Heart Rhythm Society (APHRS) AF Registry. The diagnosis of COPD was based on data reported in the case report form by the investigators. Cox‐regression models were used to assess the 1‐year risk of a primary composite outcome of all‐cause death, thromboembolic events, acute coronary syndrome, and heart failure. Analysis on single outcomes and cardiovascular death was also performed. Interaction analysis was used to assess the risk of composite outcome and all‐cause death in different subgroups. The study included 4094 patients with AF (mean±SD age 68.5±12 years, 34.6% female), of whom 112 (2.7%) had COPD. Patients with COPD showed a higher incidence of the primary composite outcome (25.1% versus 6.3%, *P*<0.001), all‐cause death (14.9% versus 2.6%, *P*<0.001), cardiovascular death (2.0% versus 0.6%, *P*<0.001), and heart failure (8.3% versus 6.0%, *P*<0.001). On multiple Cox‐regression analysis, COPD was associated with a higher risk of the primary composite outcome (hazard ratio [HR], 3.17 [95% CI, 2.05–4.90]), all‐cause death (HR, 3.59 [95% CI, 2.04–6.30]), and heart failure (HR, 3.32 [95% CI, 1.56–7.03]); no statistically significant differences were found for other outcomes. The association between COPD and mortality was significantly modified by the use of beta blockers (*P*_int_=0.018).

**Conclusions:**

In Asian patients with AF, COPD is associated with worse prognosis. In patients with AF and COPD, the use of beta blockers was associated with a lower mortality.

**Registration Information:**

clinicaltrials.gov Identifier: NCT04807049.

**Nonstandard Abbreviations and Acronyms**
APHRS: Asia‐Pacific Heart Rhythm Society
BB: beta blocker
EHRA: European Heart Rhythm Association
ESC: European Society of Cardiology
OAC: oral anticoagulation

**Clinical Perspective**

**What Is New?**

- In a prospective cohort of Asian patients with atrial fibrillation, we found that chronic obstructive pulmonary disease was associated with a frail clinical phenotype and an increased 1‐year risk of all‐cause death and heart failure.
- In this cohort, beta blockers were underprescribed, yet associated with a better outcome.

**What Are the Clinical Implications?**

- A proactive search for chronic obstructive pulmonary disease in Asian patients with atrial fibrillation might help assess the magnitude of its impact on the risk of associated adverse events and facilitate the implementation of management approaches.

Atrial fibrillation (AF) is the most common arrhythmia worldwide and is associated with a significantly increased risk of morbidity and mortality.^1^ Growing evidence shows that in patients with AF, beyond the risk of cardioembolic events, the residual risk of cardiovascular events and death, even in those already treated by oral anticoagulation (OAC), is mainly driven by the coexistence of other comorbidities.^2^, ^3^ Among the latter, chronic obstructive pulmonary disease (COPD) represents one of the most frequently detected with an estimated pooled prevalence of 13% and a significantly increased risk of hospitalization, cardiovascular events, and death.^4^, ^5^, ^6^, ^7^

The relationship between COPD and AF is complex and bidirectional. Indeed, COPD is associated with a high risk of incident AF,^8^, ^9^; facilitates the transformation of paroxysmal AF to persistent AF,^10^ and causes a high rate of recurrence after catheter ablation procedures.^11^ In addition, the onset of AF in patients with COPD is associated with a worse quality of life and an increased risk of respiratory failure.^12^

To date, the evidence supporting the increased risk of adverse events in patients with AF and COPD is largely underpinned by studies performed in Western populations and robust prospective data regarding the impact of COPD on the clinical course of AF in Asian countries is therefore much needed.

In 2015 the Asia‐Pacific Heart Rhythm Society (APHRS), in collaboration with the European Society of Cardiology, started a registry in 5 different Asian countries (Hong Kong, Singapore, South Korea, Japan, and Taiwan) to collect prospective data about the clinical course of patients with AF. The aim of this study was to evaluate the risk of adverse events in Asian patients with AF and COPD enrolled from the prospective multinational APHRS registry.

## Methods

The data that support the findings of this study are available from the corresponding author upon reasonable request.

The APHRS registry adopted the same study protocol of the European Society of Cardiology‐European Heart Rhythm Association (EHRA) EURObservational Research Programme in AF General Long‐Term (EORP‐AF) Registry.^13^, ^14^ The population was composed of consecutive in patients and outpatients with AF who had undergone a cardiology examination in tertiary and general hospitals in 5 Asian‐Pacific countries (Hong Kong, South Korea, Japan, Singapore, and Taiwan). Enrollment into the registry started in 2015, and the end of enrollment was in 2017. All eligible patients had an ECG documenting AF within 1 year before the enrollment visit. All enrolled patients signed a written informed consent according to the local regulations and the declaration of Helsinki. The study protocol was approved by the local ethics committee and was registered on ClinicalTrials.gov (NCT04807049).

At enrollment, investigators recorded each patient's clinical characteristics, comorbidities, and drugs prescribed using a standardized case report form. For this analysis, we evaluated the history of the following diseases: COPD, hypertension, coronary and peripheral artery disease, heart failure, diabetes, dyslipidemia, history of stroke or transient ischemic attack, previous bleeding, chronic kidney disease, active cancer, and dementia and the use of the following medications: angiotensin‐converting enzyme inhibitors, angiotensin receptor blockers, beta blockers (BB; without distinction between β1 selective and β1 nonselective), statins, oral antidiabetics, insulin digoxin, aldosterone blockers, calcium channel blockers (nondihydropyridine and dihydropyridine), proton pump inhibitors, and antiarrhythmics (amiodarone, flecainide, propafenone, dronedarone, and sotalol). After the enrollment, a 1‐year follow‐up was performed by the local investigators.

### Clinical Scores

The CHA_2_DS_2_‐VASc score was calculated as follows: congestive heart failure (1 point); hypertension (1 point); age 65 to 74 years (1 point) and >75 years (2 points); diabetes (1 point); stroke (2 points); vascular disease (1 point); and female sex (1 point).^15^ The HAS‐BLED score was calculated as follows: uncontrolled hypertension (1 point), abnormal renal or liver function (defined as dialysis, renal transplant, serum creatinine >200 mmol/L for the former and liver cirrhosis, bilirubin >2 × upper limit of normal, aspartate aminotransferase/alanine transaminase/alkaline phosphatase >3 × upper limit of normal for the latter, 1 point each); history of stroke (1 point); history of bleeding (1 point); labile international normalized ratio (1 point); age >65 years (1 point); and drugs (eg, aspirin or nonsteroidal anti‐inflammatory drugs) or alcohol use (1 point).^16^

Classification of AF‐related symptoms was performed according to the EHRA AF symptom classification (EHRA score)^17^ as follows: EHRA I, no symptoms; EHRA II, mild symptoms (normal daily activity not affected); EHRA III, severe symptoms (normal daily activity affected); or EHRA IV, disabling symptoms (normal daily activity discontinued).

### Study Outcomes

Adverse outcomes were recorded during 1‐year follow‐up. The primary end point of the study was time to the first occurrence of a composite of all‐cause death, any thromboembolic events, acute coronary syndrome, or significant coronary artery disease requiring percutaneous coronary intervention and new or worsening of a preexistent heart failure. Secondary outcomes were the risk of each component of the composite outcome and cardiovascular death. All‐cause death was defined as death due to cardiovascular, noncardiovascular, or unknown causes. Cardiovascular death was defined as death due to fatal cardiac (acute coronary syndromes, heart failure, arrhythmia, cardiac perforation, tamponade, or other unspecified cardiac causes) or vascular (ischemic stroke, hemorrhagic stroke, systemic hemorrhages, peripheral embolism, and pulmonary embolism) events.

### Statistical Analysis

Data were checked for normality. Categorical variables were reported as counts and percentages and compared by χ^2^ test. Continuous variables were expressed as mean±SD and compared by Student's *t* test. Univariable and multiple logistic regression analyses were used to investigate the factors associated with the prescription of BB and OAC in patients with AF. The multiple logistic regression analyses for BB and OAC prescription were adjusted for the following variables: age >75 years, female sex, paroxysmal AF, hypertension, coronary and peripheral artery disease, heart failure, diabetes, dyslipidemia, active smoking habit, previous stroke, chronic kidney disease, cancer, dementia, COPD, EHRA score (I–II versus III–IV), and previous bleeding.

The incidence rate of adverse outcomes was calculated as the number of the first occurrence of the events/total person‐years ratio and reported as incidence for 100 persons/year with relative 95% CI. Cox proportional hazards regression analysis was used to calculate the unadjusted and adjusted relative hazard ratios (HRs) and 95% CI of primary and secondary outcomes. For the primary outcome, we also reported Kaplan–Meier curves; survival distribution was compared with log‐rank test. All the multiple Cox regression analyses models were adjusted for the following covariates: age, sex, CHA_2_DS_2_‐VASc score, paroxysmal AF, chronic kidney disease, cancer, dyslipidemia, dementia, OAC, and BB use.

Additionally, we performed an interaction analysis to assess the risk of composite outcome and all‐cause death associated to COPD in relevant subgroups (age <75 years or ≥75 years, sex, paroxysmal AF, chronic kidney disease, OAC, and BB). All the interaction analyses were adjusted for the same variables used in the Cox‐regression multivariable model.

Patients lost to follow‐up or without available data about the COPD status were excluded from the analysis. All tests were 2 tailed, and analyses were performed using computer software packages (SPSS‐25.0, SPSS Inc., Chicago, IL). A *P* value <0.05 was considered as statistically significant.

## Results

Of the 4666 patients enrolled in the APHRS study, 4094 (87.7%) had available follow‐up data and information about the presence of COPD at baseline (Figure S1). Patients excluded from the analysis showed no significative differences in age (68.0±13.1 versus 68.5±11.9 years, *P*=0.309), the prevalence of female sex (30.5% versus 34.6%, *P*=0.053), mean CHA_2_DS_2_‐VASc score (2.6±1.8 versus 2.7±1.7, *P*=0.101), and mean HAS‐BLED score (1.34±1.07 versus 1.37±1.04, *P*=0.414) compared with those included in this analysis.

### Clinical Characteristics

The final cohort consisted of 4094 patients with AF (mean age 68.5±11.9 years, 34.6% female), of whom 112 (2.7%) were affected by COPD. Patients with AF and COPD were older, more often male, with more AF‐related symptoms, lower body mass index (BMI), and with a higher prevalence of heart failure, smoking habits, chronic kidney disease, dementia, and previous major extracranial bleeding (Table 1).

**Table 1:** Baseline Characteristics of Patients With Atrial Fibrillation and Chronic Obstructive Pulmonary Disease

Characteristics	COPD, n=112	Non‐COPD, n=3982	*P* value
Age, y, mean±SD	75.8±11.2	68.3±11.8	<0.001
Body mass index, k/m^2^	23.6±4.1	25.1±4.2	0.001
Female sex, n (%)	14 (12.5)	1404 (35.3)	<0.001
Atrial fibrillation pattern, n (%)			
First diagnosed	5 (4.5)	286 (7.2)	0.673
Paroxysmal	45 (40.2)	1664 (41.9)	
Persistent	26 (23.2)	945 (23.8)	
Long‐standing persistent	14 (12.5)	397 (10.0)	
Permanent	22 (19.6)	677 (17.1)	
Concomitant disease, n (%)			
Hypertension	71 (65.1)	2425 (61.2)	0.408
Coronary artery disease	29 (26.1)	756 (19.3)	0.072
Heart failure	40 (36.4)	814 (20.7)	<0.001
Diabetes	28 (25.2)	965 (24.6)	0.883
Dyslipidemia	34 (30.4)	1513 (38.6)	0.075
Smoker	15 (13.4)	327 (8.2)	0.051
Previous stroke/transient ischemic attack	14 (12.6)	378 (9.6)	0.287
Previous bleedings	13 (11.6)	296 (7.5)	0.105
Intracranial hemorrhage	2 (1.8)	68 (1.7)	0.958
Major extracranial bleeding	8 (7.1)	121 (3.1)	0.015
Peripheral artery disease	1 (0.9)	51 (1.3)	0.729
Chronic kidney disease	14 (12.5)	297 (7.5)	0.047
Cancer	3 (2.7)	92 (2.3)	0.799
Dementia	5 (4.5)	68 (1.7)	0.030
Medications, n (%)			
Angiotensin‐converting enzyme inhibitors	18 (16.2)	518 (13.1)	0.330
Angiotensin receptor blockers	33 (29.7)	1036 (26.1)	0.391
Beta blockers	44 (39.6)	2025 (51.1)	0.017
Statins	39 (34.2)	1503 (37.9)	0.429
Oral antidiabetics	16 (14.4)	638 (16.1)	0.633
Insulin	3 (2.7)	99 (2.5)	0.890
Digoxin	10 (8.9)	450 (11.3)	0.427
Diuretics	18 (16.1)	881 (22.2)	0.123
Aldosterone blockers	13 (11.6)	256 (6.4)	0.080
Calcium channel blockers	24 (21.4)	930 (23.4)	0.619
Proton pump inhibitors	32 (28.6)	1142 (28.7)	0.832
Antiarrhythmics	26 (23.4)	898 (22.6)	0.491
Amiodarone	11 (9.8)	312 (7.9)	0.449
Flecainide	6 (5.4)	178 (4.5)	0.660
Propafenone	8 (7.2)	282 (7.1)	0.966
Dronedarone	2 (1.8)	98 (2.5)	0.645
Sotalol	0 (0.0)	72 (1.8)	0.151
Symptomatic status, n (%)			
EHRA score I	72 (64.3)	2561 (64.3)	0.007
EHRA score II	26 (23.2)	1175 (29.5)	
EHRA score III	14 (12.5)	216 (5.4)	
EHRA score IV	30 (0.8)	0 (0.0)	
Thrombotic and hemorrhagic risk			
CHA_2_DS_2_‐VASC score, mean±SD	3.2±1.5	2.7±1.7	0.002
CHA_2_DS_2_‐VASC score ≥2, n (%)	101 (90.2)	2892 (72.6)	<0.001
HAS‐BLED score, mean±SD	1.5±1.1	1.1±0.9	<0.001
HAS‐BLED score ≥3, n (%)	22 (19.6)	543 (13.6)	0.069
Oral anticoagulation, n (%)	94 (83.9)	3283 (82.4)	0.684
Non‐vitamin K antagonist, n (%)	67 (71.3)	2487 (75.8)	
Vitamin K antagonist, n (%)	27 (28.7)	796 (24.2)	0.319
Antiplatelets, n (%)	18 (16.1)	596 (15.0)	0.747

COPD indicates chronic obstructive pulmonary disease; and EHRA, European Heart Rhythm Association AF symptom classification.

**Figure 1:**
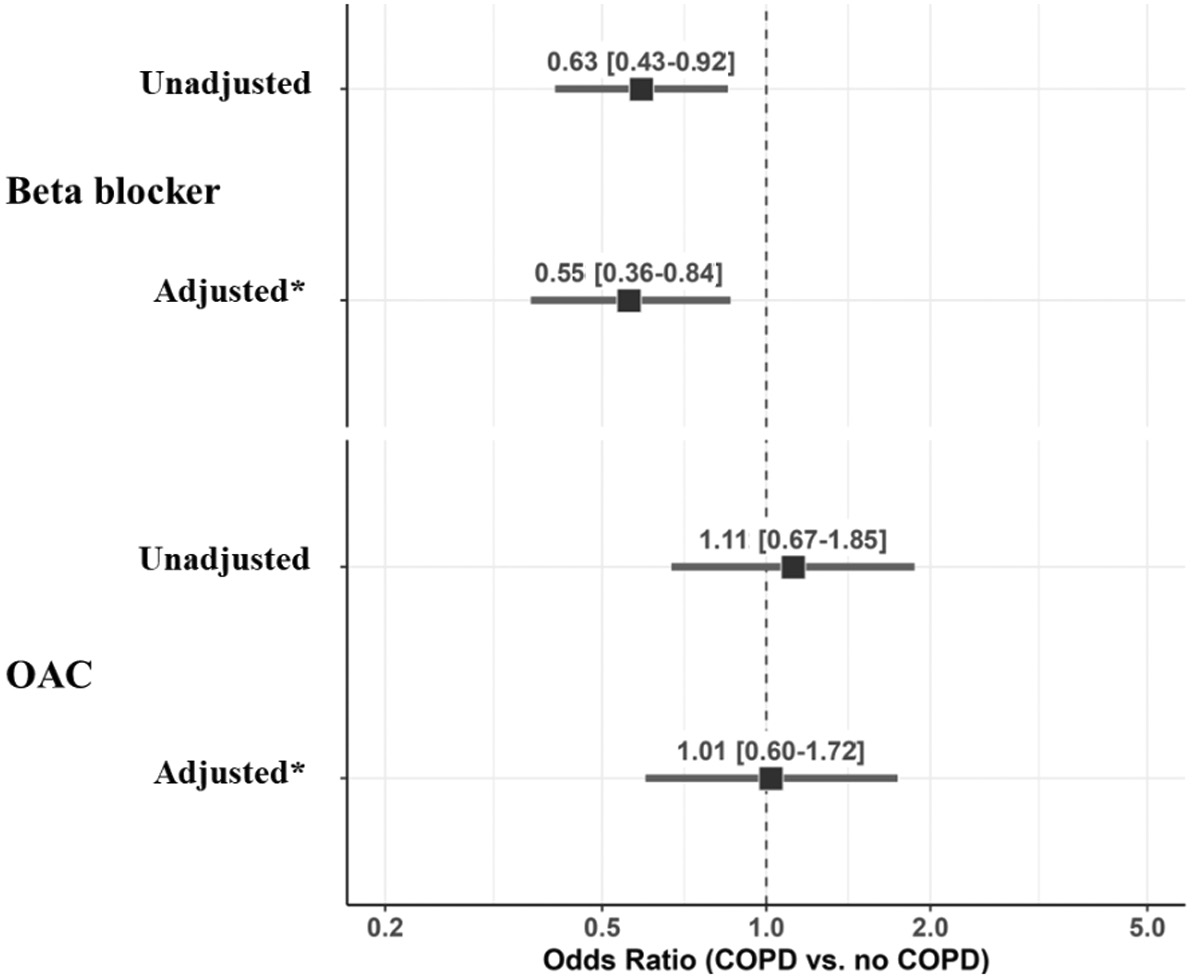
Univariable and multivariable logistic regression analyses for beta blocker and oral anticoagulation prescription in patients with chronic pulmonary obstructive disease. *Multivariable logistic regression analysis adjusted for age ≥75 years, female sex, paroxysmal atrial fibrillation, hypertension, coronary artery disease, heart failure, diabetes, dyslipidemia, smoking habits, previous stroke/transient ischemic attack, peripheral artery disease, chronic kidney disease, cancer, dementia, European Heart Rhythm Association score, and previous bleeding. COPD indicates chronic obstructive pulmonary disease; and OAC, oral anticoagulation.

A significantly lower use of BB was found in patients with COPD compared with those without COPD, whereas no differences were detected for OAC use, OAC type, antiplatelet drugs, and other medications (Table 1). The lower use of BB in patients with COPD was also demonstrated by univariable (odds ratio [OR], 0.63 [95% CI, 0.43–0.92]) and multivariable logistic regression analysis (OR, 0.55 [95% CI, 0.36–0.84]) after adjustment for confounding factors (Figure 1; Table S1). Logistic regression analyses performed to investigate the clinical factors associated with OAC prescription did not show any significant associations with COPD in both the univariable (OR, 1.11 [95% CI, 0.67–1.85]) and multivariable analyses (OR, 1.01 [95% CI, 0.60–1.72]; Figure 1; Table S2).

### Follow‐Up and Survival Analysis

After 1‐year follow‐up, the following adverse events were recorded: 267 (6.5%) composite outcomes, 118 (2.9%) all‐cause deaths, 26 (0.6%) cardiovascular deaths, 28 (0.7%) thromboembolic events, 41 (1.0%) acute coronary syndromes, and 94 (2.3%) new or worsening heart failure. Compared with patients with AF without COPD, those with COPD had significantly higher incidence rates (IRs) of the composite outcome (IR per 100 patient‐years, 6.3 [95% CI, 5.5–7.2] versus 25.1 [16.1–37.3], *P*<0.001), all‐cause death (IR per 100 patient‐years 2.6 [95% CI, 2.1–3.2] versus 14.9 [95% CI, 8.3–24.5], *P*<0.001), cardiovascular death (IR per 100 patient‐years, 0.6, [95% CI, 0.4–0.9] versus 2.0 [95% CI, 0.2–7.2], *P*<0.001), and new or worsening heart failure (IR per 100 patient‐years, 6.0 [95% CI, 2.9–9.1] versus 8.3 [95% CI, 3.6–16.3], *P*<0.001) (Table 2). No significantly different incidence rates were found for other outcomes.

**Table 2:** Incidence Rates and Cox Regression Analyses For Risk of Primary and Secondary Outcomes According to COPD

	Number of events	Incidence rate 100 patient‐y (95% CI)	*P* value	Univariable analysis HR (95% CI)	Multivariable analysis* HR (95% CI)
Composite outcome					
COPD	24	25.1 (16.1–37.3)	<0.001	3.96 (2.61–6.03)	3.17 (2.05–4.90)
No COPD	243	6.3 (5.5–7.2)		Reference	Reference
All‐cause death					
COPD	15	14.9 (8.3–24.5)	<0.001	5.64 (3.28–9.70)	3.59 (2.04–6.30)
No COPD	103	2.6 (2.1–3.2)		Reference	Reference
Cardiovascular death					
COPD	2	2.0 (0.2–7.2)	<0.001	3.24 (0.77–13.70)	2.65 (0.60–11.70)
No COPD	24	0.6 (0.4–0.9)		Reference	Reference
Thromboembolic events					
COPD	1	1.0 (0.3–5.5)	0.718	1.51 (0.21–11.20)	1.48 (0.19–11.48)
No COPD	27	0.7 (0.5–1.0)		Reference	Reference
Acute coronary syndrome/percutaneous coronary intervention					
COPD	1	1.0 (0.2–5.5)	0.979	0.96 (0.13–6.97)	0.72 (0.10–5.36)
No COPD	40	1.0 (0.7–1.4)		Reference	Reference
New or worsening heart failure					
COPD	8	8.3 (3.6–16.3)	<0.001	3.52 (1.71–7.26)	3.32 (1.56–7.03)
No COPD	88	6.0 (2.9–9.1)		Reference	Reference

COPD indicates chronic obstructive pulmonary disease; and HR, hazard ratio.

* Adjusted for: age, sex, paroxysmal atrial fibrillation, CHA_2_DS_2_‐VASc score, chronic kidney disease, cancer, dyslipidemia, dementia, oral anticoagulation, and beta blocker use.

**Figure 2:**
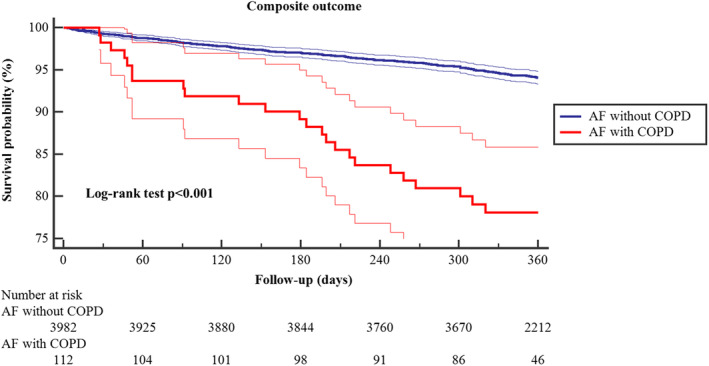
Kaplan–Meier curve for the risk of composite outcome in patients with atrial fibrillation and chronic obstructive pulmonary disease. Thick lines represent survival probability, and thin lines represent 95% CIs. AF indicates atrial fibrillation; and COPD, chronic obstructive pulmonary disease.

Kaplan–Meier curves for the primary composite outcome are shown in Figure 2 with a lower survival probability for patients with AF and COPD (log‐rank test *P*<0.001).

On univariable Cox‐regression analysis, COPD was associated with a higher risk of the primary composite outcome (HR, 3.96 [95% CI, 2.61–6.03]), all‐cause death (HR, 5.64 [95% CI, 3.28–9.70]), and new or worsening heart failure (HR, 3.52 [95% CI, 1.71–7.26]). No significant associations were found for cardiovascular death, acute coronary syndromes, and thromboembolic events (Table 2).

On multivariable Cox‐regression analysis, COPD was still associated with a higher risk of composite outcome (HR, 3.17 [95% CI, 2.05–4.90]), all‐cause death (HR, 3.59 [95% CI, 2.04–6.30]), and new or worsening heart failure (HR, 3.32 [95% CI, 1.56–7.03]) (Table 2; Tables S3 through S5).

### Subgroup Analysis

**Figure 3:**
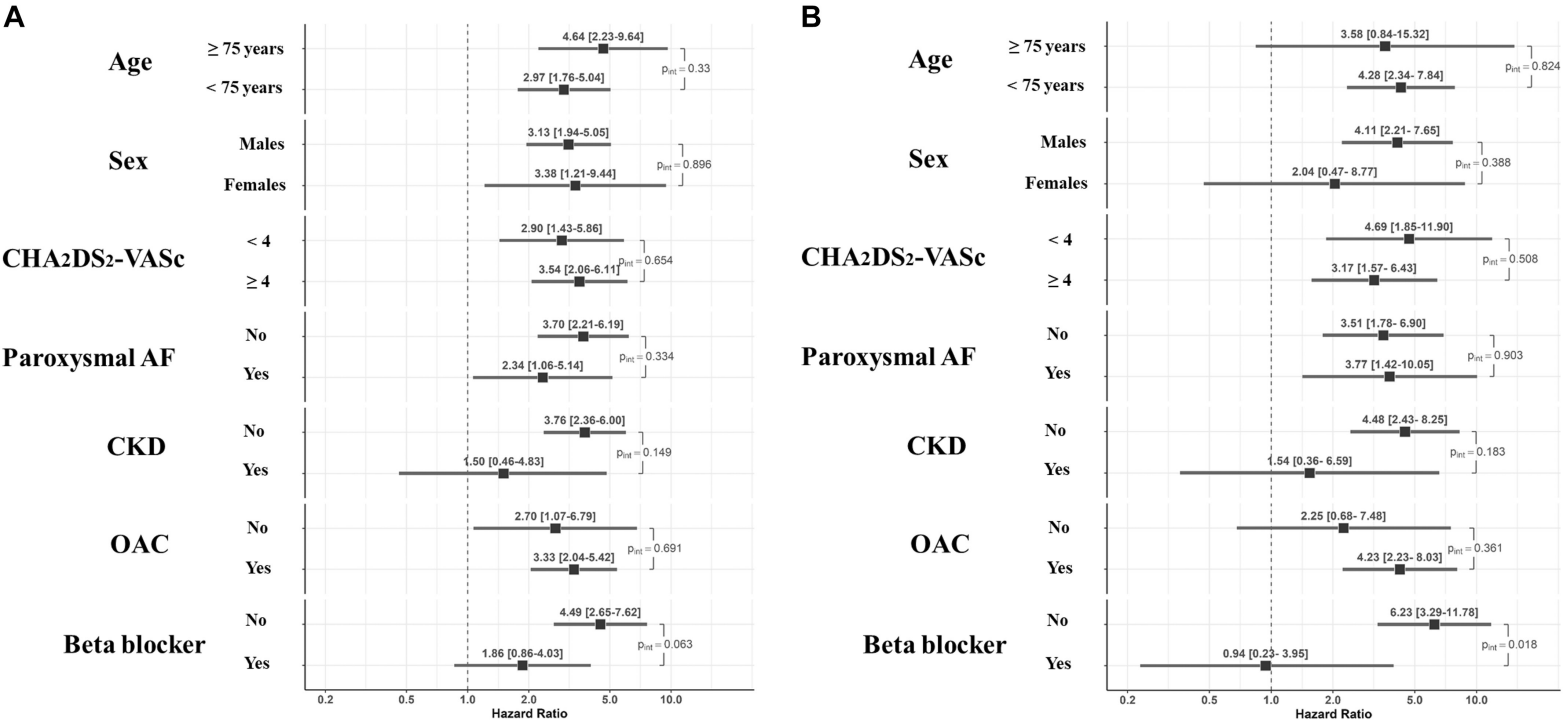
Risk of composite outcome (A) and all‐cause death (B) in patients with chronic obstructive pulmonary disease according to different subgroups. AF indicates atrial fibrillation; CKD, chronic kidney disease; and OAC, oral anticoagulation.

No statistically significant interaction was found for the risk of the primary composite outcome in patients with COPD with age (<75 years or ≥75 years; *P*_int_=0.330), sex (*P*_int_=0.896), CHA_2_DS_2_‐VASc score <4 versus ≥4 (*P*_int_=0.654), paroxysmal AF (versus all other AF types; *P*_int_=0.334), chronic kidney disease (*P*_int_=0.149), and OAC (*P*_int_=0.691), but a nonsignificant trend for a lower risk was found according to the prescription of BB at baseline (*P*_int_=0.063; Figure 3A).

Analyzing the interaction between COPD and the risk of all‐cause death, we found no statistically significant differences for age (*P*_int_=0.824), sex (*P*_int_=0.388), CHA_2_DS_2_‐VASc score <4 versus ≥4 (*P*_int_=0.508), paroxysmal AF (*P*_int_=0.903), chronic kidney disease (*P*_int_=0.183), and OAC (*P*_int_=0.361). However, the association between COPD and mortality was found to be significantly modified by the use of BB (*P*_int_=0.018) (Figure 3B).

## Discussion

In our study on Asian patients with AF from the APHRS registry, our principal findings are as follows: (1) the prevalence of COPD was 2.7%; (2) COPD was associated with a clinical phenotype characterized by low BMI, high prevalence of male sex, smoking habits, heart failure, chronic kidney disease, dementia, and previous major extracranial bleeding; (3) COPD was associated with a high 1‐year risk of composite outcome, all‐cause death, and heart failure, after adjustment for confounding factors; and (4) the association between COPD and the risk of all‐cause death appeared modified by the use of BB.

The COPD prevalence of about 3% that we found in our Asian population with AF was significantly lower compared with the 11% reported in the EORP‐AF registry conducted in an European cohort^6^ and also lower compared with the pooled prevalence of 9.4% found by a recent metanalysis in Asian populations with AF.^4^ Possible explanations for these differences include the different predisposing factors in Western and Asian patients, the different study designs, and the possibly different COPD definitions. Indeed, in Western countries the COPD diagnosis is generally based on lung function tests, whereas in Asian countries, COPD is often assessed through the self‐reported history of respiratory symptoms, thus possibly leading to underdiagnosis of COPD, which may have contributed to the low COPD prevalence in our study.^18^ The issue of COPD underdiagnosis in Asians was highlighted by a large epidemiologic study performed in Japan, whereby 90% of patients found with an obstructive irreversible pattern on spirometry test did not have a prior diagnosis of COPD.^19^

In our cohort of Asian patients with AF, we found that those with COPD were more often male, smokers, with low BMI, and with a high prevalence of previous major bleeding. It is well known that smoking habits are associated with COPD risk, as well as male sex and low BMI.^20^ However, the association between smoking and low BMI in the present study could be partly responsible for the high prevalence of previous bleeding found in patients with COPD. Indeed, in Asians with AF who were anticoagulated, low body weight could be associated with alterations in OAC metabolism and a higher risk of bleeding.^21^

When analyzing the 1‐year risk of adverse events, we showed that the high risk of composite outcome in patients with AF and COPD was driven mainly by an increased risk of all‐cause death and heart failure. These results are consistent with previous evidence showing that COPD is associated with higher mortality in Asian patients with AF, independently from other possible confounding factors. For example, in 352 656 Taiwanese patients with AF (COPD prevalence 35.5%) from its National Health Insurance Research Database, COPD was associated with a significantly higher risk of ventricular arrhythmias or sudden cardiac death when compared with patients without COPD (HR, 1.21 [95% CI, 1.16–1.25]), after adjustment for age, sex, heart failure, hypertension, diabetes, previous stroke, vascular disease, chronic kidney disease, cancer, autoimmune disease, and liver cirrhosis.^22^ In a multicenter study of 1975 AF Chinese patients attending the emergency department (COPD prevalence, 11.5%), the presence of COPD was associated with a high risk of all‐cause death (HR, 1.49 [95% CI, 1.11–2.00]) and cardiovascular death (HR, 1.60 [95% CI, 1.07–2.38]) after adjustment for age, body weight, AF pattern, heart rate, heart failure, diabetes, and previous stroke.^23^

Furthermore, our findings are in line with the high risk of all‐cause death (OR, 2.22 [95% CI, 1.93–2.55]) shown by a meta‐analysis on more than 4 000 000 patients with AF from both Western and Asian populations,^4^ thus confirming that COPD in patients with AF is associated with a high risk of mortality independent of the geographical area considered.

The pathophysiological mechanisms by which COPD increases the risk of all‐cause death in patients with AF are complex and involve multiple factors. The chronic hypoxemia and hypercapnia associated with COPD induce vasoconstriction and pulmonary hypertension and could lead to right ventricular failure.^24^ The hypoxemia observed in patients with COD is responsible for a systemic proinflammatory state that could precipitate thrombotic events.^25^ Moreover, the sympathetic stimulation that occurs during the COPD exacerbations, beyond its relaxing effect on the airways' smooth muscle cells, could have detrimental effects on the cardiocirculatory system facilitating myocardial ischemic events and life‐threatening ventricular arrhythmias.^26^

These different effects mediated by beta receptors on the lung and heart system have raised theoretical concerns on the use of BB in patients with COPD and AF, that still mitigate their wider use in clinical practice.^27^ Indeed, although several studies showed that BB use in patients with COPD and cardiovascular disease was associated with a reduced risk of death and did not interact with the bronchodilators' action but prevented their protachycardic effect,^28^ in our cohort of patients with AF we found a significant underprescription of this treatment. The importance of this finding was further underlined by the demonstration that the lack of a BB therapy was associated with a worse prognosis, thus providing the first evidence of the beneficial effect of BB therapies in Asian patients with AF and COPD derived from a large, multinational, and prospective cohort.

### Limitations

When interpreting the results of this study there are some limitations to consider. First, this is an observational study, and no causality effect could be derived from our results for the possible presence of selection bias, reporting bias, and unmeasured confounding. Second, our study population is mainly composed of patients from high‐income countries and does not include the Indonesian and Pacific Islander populations, thus providing only a partial scenario of the wide Asian heterogeneity. Third, although the difference between patients excluded and included in this analysis seems to be minimal, some residual bias could have been associated with the exclusion of patients without follow‐up or COPD status. Fourth, the diagnosis of COPD was based on the data reported on the clinical research form and no lung function tests were performed to assess the presence or the severity of the obstructive pulmonary pattern. Finally, the low COPD prevalence and the small number of adverse events recorded after 1‐year follow‐up may have affected our statistical analysis making it unable to detect significant differences.

Notwithstanding these limitations, we found a strong association between COPD and the risk of all‐cause death and heart failure in a contemporary multinational cohort of patients with AF, reinforcing the recommendations about the importance of early COPD recognition in patients with AF.^29^ Indeed, a proactive search for COPD in Asian patients with AF might help not only to estimate its real prevalence but most important to assess the magnitude of its impact on the risk of associated adverse events and facilitate implementation of management approaches. Indeed, COPD should be part of the spectrum of comorbidities that should be proactively managed, in the current guideline‐recommended holistic or integrated care approach to AF.^30^ Adherence with the latter approach has been associated with improved clinical outcomes.^31^, ^32^

## Conclusions

In this large prospective cohort of Asian patients with AF, COPD is consistently associated with worse prognosis. The use of BB in patients with AF and COPD was associated with a lower risk of all‐cause mortality, supporting the use of BB when indicated in COPD.

### Appendix

APHRS‐AF Registry Investigators

Hong Kong: Chun‐Wah Siu David (Queen Mary Hospital). Japan: Wataru Shimizu, Kenji Yodogawa (Department of Cardiovascular Medicine, Medical School); Hiroyuki Tsutsui, Yasushi Mukai (Department of Cardiovascular Medicine, Faculty of Medical Sciences, Kyushu University); Hirofumi Tomita, Daisuke Horiuchi (Department of Cardiology, Hirosaki University Graduate School of Medicine); Joji Hagii (Hirosaki Stroke and Rehabilitation Center); Kazutaka Aonuma (Division of Cardiology, University of Tsukuba Hospital); Yasuo Okumura (Division of Cardiology, Nihon University, Itabashi Hospital); Masahiko Goya, Kenzo Hirao (Department of Cardiovascular Medicine, Tokyo Medical and Dental University); Ajioka (Division of Cardiology, Tosei General Hospital); Nobuhisa Hagiwara, Atsushi Suzuki (Department of Cardiology, Tokyo Women's Medical University); Teiichi Yamane (Department of Cardiovascular Medicine, Jikei University); Takanori Ikeda, Hitomi Yuzawa (Toho University, Faculty of Medicine); Kazuhiro Satomi, Yoshinao Yazaki (Heart Rhythm Center, Tokyo Medical University); Keiichi Fukuda (Department of Cardiology, Keio University School of Medicine); Yoshinori Kobayashi, Norishige Morita (Division of Cardiology, Tokai University Hachioji‐hospital); Toyoaki Murohara (Department of Cardiology, Nagoya University); Eiichi Watanabe, Masahide Harada (Department of Cardiology, Fujita Health University School of Medicine); Satoru Sakagami, Takahiro Saeki (National Hospital Organization, Kanazawa Medical Center); Kengo Kusano, Koji Miyamoto (Department of Cardiovascular Medicine, National Cerebral and Cardiovascular Center); Shinsuke Miyazaki, Hiroshi Tada (Department of Cardiovascular Medicine, University of Fukui); Koichi Inoue, Nobuaki Tanaka (Cardiovascular center, Sakurabashi Watanabe Hospital); Yukihiro Koretsune, Haruhiko Abe (National Hospital Organization Osaka National Hospital); Yasuki Kihara, Yukiko Nakano (Department of Cardiovascular Medicine, Hiroshima University); Akihiko Shimizu, Yasuhiro Yoshiga (Department of Medicine and Clinical Science, University Graduate School of Medicine); Tomohiro Sakamoto, Ken Okumura (Division of Cardiology, Saiseikai Kumamoto Hospital Cardiovascular Center); Naohiko Takahashi, Tetsuji Shinohara (Oita University Hospital); Kyoko Soejima (Department of Cardiovascular Medicine, Kyorin University School of Medicine); Masahiko Takagi (Kansai Medical University Medical Center); Mitsuharu Kawamura, Yumi Munetsugu (Division of Cardiology, Showa University School of Medicine). Korea: Sung‐Hwan Kim (Division of Cardiology, The Catholic University of Korea); Jae‐Min Shim (Division of Cardiology, Korea University College of Medicine and Korea University Medical Center); Jae Sun Uhm (Division of Cardiology, Yonsei University College of Medicine); Sung Il Im (Division of Cardiology, Kosin University College of Medicine); Hyoung‐Seob Par (Division of Cardiology, Department of Internal Medicine, Keimyung University Dongsan Hospital); Jun Hyung Kim (Department of Cardiology, Chungnam National University); Young Keun On (Division of Cardiology, Sungkyunkwan University School of Medicine); Il‐Young Oh (Division of Cardiology Seoul National University Bundang Hospital); Seung Yong Shin (Cardiovascular & Arrhythmia Centre, Chung‐Ang University); Jum Suk Ko (Division of Cardiology, Department of Internal Medicine, Wonkwang University School of Medicine, Iksan, Korea); Jun Beom Park (Department of Cardiology, College of Medicine, Ewha Womans University, Seoul, Korea). Singapore: Wee‐Siong Teo (National Heart Centre Singapore); Kelvin Cheok‐Keng Wong (Changi General Hospital); Toon‐Wei Lim (National University Hospital); David Foo (Tan Tock Seng Hospital). Taiwan: Shih‐Ann Chen (Taichung Veterans General Hospital); Shih‐Ann Chen, Tze‐Fan Chao, YennJiang Lin, Fa‐Po Chung, Yu‐Feng Hu, Shil‐Lin Chang, Ta‐Chuan Tuan, Jo‐Nan Liao (Taipei Veterans General Hospital); Cheng‐Hung Li, Jin‐Long Huang, Yu‐Cheng Hsieh, Tsu‐Juey Wu, Ying‐Chieh Liao (Taichung Veterans General Hospital); Cheng‐Hung Chiang, Hsiang‐Chiang Hsiao, Tung‐Chen Yeh (Kaohsiung Veterans General Hospital); Wei‐Siang Lin, Wen‐Yu Lin (Tri‐Service General Hospital); Jen‐Yuan Kuo, Chong‐Lie Hong, Yih‐Je Wu, Ying‐Siang Li, Jui‐Peng Tsai, Kuo‐Tzu Sung, Sheng‐Hsiung Chang (Mackay Memorial Hospital).

## Sources of Funding

This study was an independent research grant by Pfizer and Bristol Myers Squibb (BMS) to Asia‐Pacific Heart Rhythm Society.

## Disclosures

Giulio Francesco Romiti reports consultancy for Boehringer Ingelheim and an educational grant from Anthos, outside the submitted work; no fees are directly received personally. Wataru Shimizu has received grants from Daiichi Sankyo Co., Ltd. and Nippon Boehringer Ingelheim Co., Ltd.; and remuneration for lectures, presentations, speakers' bureaus, manuscript writing, or educational events from Daiichi Sankyo Co., Ltd., Nippon Boehringer Ingelheim Co., Ltd., Bristol‐Meyers Squibb, Bayer Yakuhin, Ltd., Pfizer Japan, Inc., Ono Pharmaceutical Co., Ltd., and Medtronic Japan Co., Ltd. Hung‐Fat Tse is a consultant/has received speaker fees and research grants from for Abbott, Amgen, AstraZeneca, Bayer, Bristol‐Meyers Squibb, Boehringer Ingelheim, Boston Scientific, Daiichi Sankyo, Medtronic, Novartis, Pfizer, and Sanofi. Marco Proietti is national leader of the AFFIRMO project on multimorbidity in AF, which has received funding from the European Union's Horizon 2020 research and innovation program under grant agreement No 899871. Gregory Y.H. Lip is a consultant and speaker for Bristol‐Meyers Squibb/Pfizer, Boehringer Ingelheim, Anthos and Daiichi‐Sankyo; no fees are received personally. Gregory Y.H. Lip is a National Institute for Health and Care Research Senior Investigator and co‐principal investigator of the AFFIRMO project on multimorbidity in AF, which has received funding from the European Union's Horizon 2020 research and innovation program under grant agreement No 899871. The remaining authors have no disclosures to report.

## Supporting information

Tables S1–S5. Figure S1.
