# Supplementary material for: Risk of Death and Cardiovascular Events in Asian Patients With Atrial Fibrillation and Chronic Obstructive Pulmonary Disease: A Report From the Prospective APHRS Registry
Source: J Am Heart Assoc. 2024 Mar 27;13(7):e032785. doi: 10.1161/JAHA.123.032785 (PMC11179754; doi:10.1161/JAHA.123.032785)

Supplemental Material

Table S1. Logistic regression analysis for the risk of beta blocker use.

	Univariable OR (95%CI)	Multivariable* OR (95%CI)
Age>75 years	0.86 (0.75-0.98)	0.69 (0.60-0.81)
Female sex	1.06 (0.93-1.21)	1.09 (0.95-1.26)
Paroxysmal atrial fibrillation	0.72 (0.64-0.82)	0.86 (0.75-0.98)
HERA score (I - II vs III - IV)	1.20 (0.93-1.55)	1.16 (0.88-1.53)
Hypertension	1.79 (1.58-2.04)	1.67 (1.45-1.93)
Coronary artery disease	1.45 (1.24-1.69)	1.17 (0.98-1.39)
Heart failure	2.29 (1.96-2.69)	2.21 (1.85-2.63)
Diabetes	1.40 (1.21-1.61)	1.14 (0.97-1.34)
Dyslipidemia	1.73 (1.52-1.96)	1.42 (1.22-1.64)
Smoker	1.09 (0.88-1.37)	1.15 (0.89-1.45)
Previous Stroke/Transient ischemic attack	1.13 (0.92-1.39)	1.08 (0.86-1.36)
Peripheral artery disease	0.80 (0.46-1.39)	0.52 (0.28-0.96)
Chronic kidney disease	1.81 (1.42-2.30)	1.59 (1.22-2.09)
Cancer	0.83 (0.55-1.25)	0.97 (0.62-1.51)
Dementia	0.84 (0.53-1.34)	0.73 (0.44-1.24)
Chronic obstructive pulmonary disease	0.63 (0.43-0.92)	0.55 (0.36-0.84)
Previous bleeding	0.83 (0.66-1.05)	0.69 (0.53-0.90)

OR: Odds Ratio, CI: Confidence of Interval.

Table S2. Logistic regression analysis for OAC use.

	**Univariable OR (95%CI)**	**Multivariable* OR (95%CI)**
Age>75 years	1.26 (1.06-1.51)	1.20 (0.98-1.47)
Female sex	1.29 (1.09-1.54)	1.29 (1.07-1.56)
Paroxysmal atrial fibrillation	0.68 (0.58-0.80)	0.74 (0.62-0.88)
HERA score (I - II vs III - IV)	1.01 (0.73-1.41)	0.93 (0.65-1.31)
Hypertension	1.56 (1.32-1.83)	1.50 (1.25-1.80)
Coronary artery disease	1.04 (0.84-1.28)	0.88 (0.71-1.11)
Heart failure	1.40 (1.13-1.74)	1.37 (1.08-1.73)
Diabetes	1.36 (1.11-1.65)	1.24 (0.99-1.54)
Dyslipidemia	1.31 (1.11-1.56)	1.10 (0.91-1.33)
Smoke	0.98 (0.73-1.30)	1.12 (0.82-1.53)
Previous Stroke/Transient ischemic attack	2.87 (1.95-4.22)	3.36 (2.19-5.17)
Peripheral artery disease	0.89 (0.45-1.79)	0.86 (0.41-1.78)
Chronic kidney disease	0.70 (0.53-0.92)	0.56 (0.41-0.76)
Cancer	0.74 (0.45-1.21)	0.72 (0.42-1.21)
Dementia	0.60 (0.35-1.02)	0.31 (0.17-0.55)
Chronic obstructive pulmonary disease	1.11 (0.67-1.85)	1.01 (0.60-1.72)
Previous bleeding	0.69 (0.52-0.91)	0.53 (0.39-0.72)

OR: Odds Ratio, CI: Confidence of Interval.

Table S3. Multiple Cox-regression analysis for composite outcome.

	HR	95%CI
Age	1.03	1.01-1.04
Female sex	0.83	0.63-1.09
Paroxysmal atrial fibrillation	0.65	0.50-0.85
CHA_2DS_2-VASC	1.33	1.21-2.32
Chronic obstructive pulmonary disease	3.17	2.05-4.90
Chronic kidney disease	1.69	1.23-2.32
Cancer	2.01	1.24-3.26
Dyslipidemia	1.43	1.11-1.85
Dementia	1.75	1.08-2.84
Oral anticoagulation	0.51	0.39-0.68
Beta blocker	1.23	2.05-4.90

HR: Hazard Ratio, CI: Confidence of Interval.

Table S4. Multiple Cox-regression analysis for all-cause death.

	HR	95%CI
Age	1.06	1.04-1.09
Female sex	0.67	0.44-1.01
Paroxysmal atrial fibrillation	0.53	0.35-0.81
CHA_2DS_2-VASC	1.46	1.27-1.68
Chronic obstructive pulmonary disease	3.59	2.04-6.30
Chronic kidney disease	1.80	1.16-2.80
Cancer	3.05	1.71-5.46
Dyslipidemia	1.31	0.89-1.92
Dementia	1.65	0.88-3.08
Oral anticoagulation	0.49	0.30-0.68
Beta blocker	1.04	0.71-1.51

HR: Hazard Ratio, CI: Confidence of Interval.

Table S5. Multiple Cox-regression analysis for new or worsening heart failure.

	HR	95%CI
Age	1.03	0.98-1.03
Female sex	0.96	0.61-1.50
Paroxysmal atrial fibrillation	0.50	0.31-0.80
CHA_2DS_2-VASC	1.35	1.15-1.58
Chronic obstructive pulmonary disease	3.32	1.56-7.03
Chronic kidney disease	1.91	1.13-3.24
Cancer	1.41	0.51-3.87
Dyslipidemia	1.32	0.86-2.02
Dementia	1.88	0.80-4.37
Oral anticoagulation	0.87	0.50-1.50
Beta blocker	3.32	1.56-7.03

HR: Hazard Ratio, CI: Confidence of Interval.

Figure S1. Patient flow of the study.

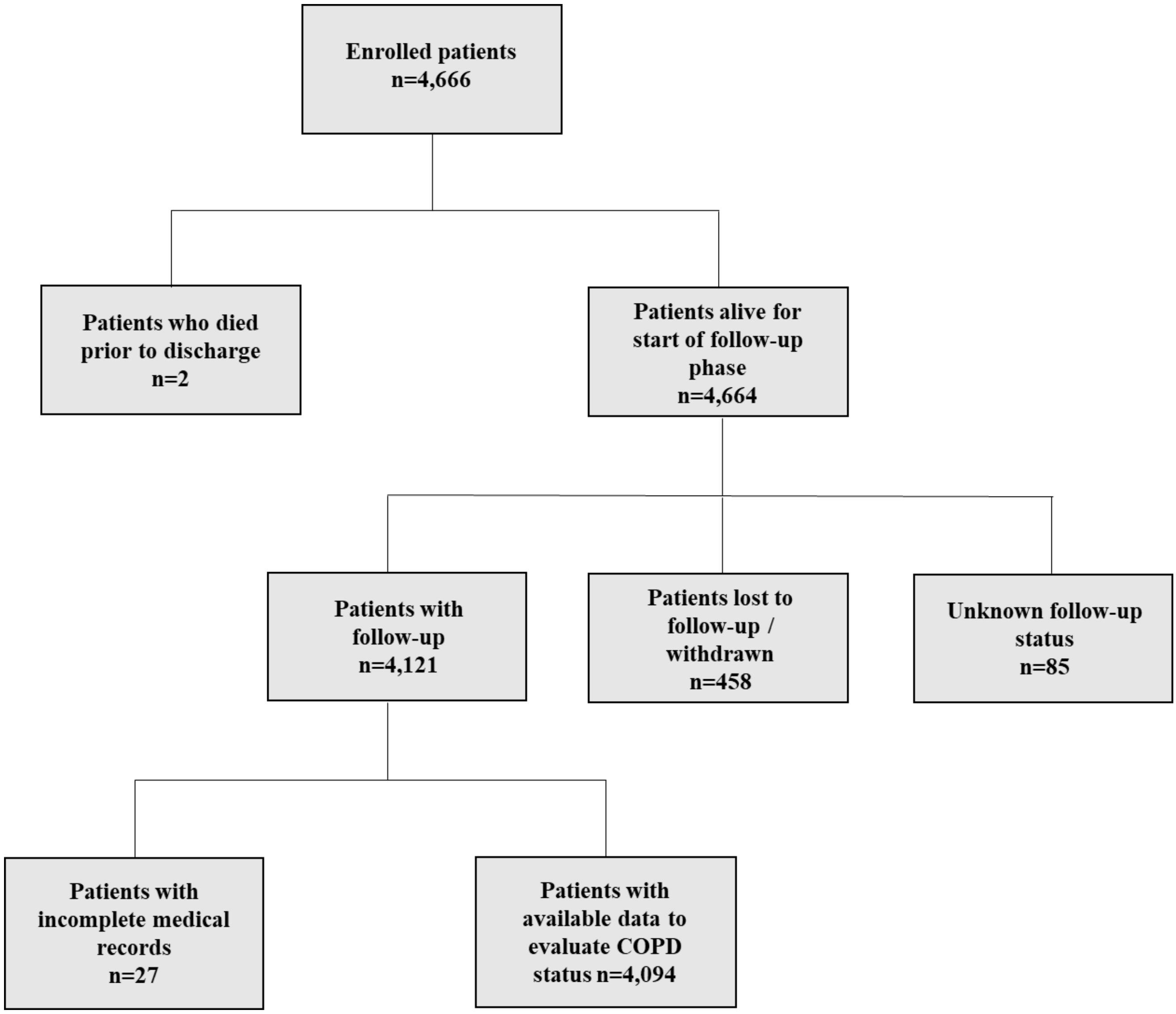

Supplement: Supplementary file 1 — Tables S1–S5. Figure S1. [file JAH3-13-e032785-s001.pdf]
